# Efficacy of the SuperTowel^®^: An Alternative Hand-washing Product for Humanitarian Emergencies

**DOI:** 10.4269/ajtmh.18-0860

**Published:** 2019-03-11

**Authors:** Belen Torondel, Rummana Khan, Torben Holm Larsen, Sian White

**Affiliations:** 1 Department of Disease Control, London School of Hygiene and Tropical Medicine, London, United Kingdom;; 2 Department of Microbiology, Kelkar Education Trust’s Scientific Research Centre, Mumbai, India;; 3 Real Relief ApS, Essen, Denmark

## Abstract

Handwashing with soap reduces the transmission of diarrheal pathogens, but access to hand-washing facilities, water, and soap in humanitarian emergencies is limited. The SuperTowel^®^ (ST) is a fabric treated with permanent antimicrobial bonding and has been designed as a soap alternative in emergency situations. The aim of this study was to test the efficacy of the ST as a hand-cleaning product. Two sets of laboratory tests, with 16 volunteers in each, were conducted to test the efficacy of different prototypes of the ST. Volunteers pre-contaminated their hands with nonpathogenic *Escherichia coli*. Comparisons were made between hand cleaning with the ST and handwashing with the reference soap, using a crossover design. Participants also completed a questionnaire about product perceptions. Three of the prototypes of the ST were more efficacious at removing *E. coli* from pre-contaminated hands than handwashing with soap (mean log_10_ reduction of 4.11 ± 0.47 for ST1, 3.84 ± 0.61 for ST2, and 3.71 ± 0.67 for ST3 versus 3.01 ± 0.63 for soap [*P* < 0.001, *P* = 0.002, and *P* = 0.005, respectively]). The ST prototypes used less water than handwashing with soap, were well accepted, and were considered preferable in communal settings. The ST has the potential to be a suitable complementary hand-cleaning product for humanitarian emergencies.

## INTRODUCTION

### Hand hygiene in emergencies.

In the wake of an emergency, populations often find themselves displaced and living in crowded conditions, with poor sanitation and hygiene facilities and a limited amount of water. These conditions create an ideal environment for the spread of communicable diseases.^1^ Of particular concern are communicable diseases that transmit through the fecal–oral route. Fecal–oral pathogens can cause diarrheal diseases, some respiratory infections, and many outbreak-related diseases (e.g., cholera). These diseases are a leading cause of preventable illness and death across all types of humanitarian crises.^2^ Handwashing with soap is one of the most effective preventative strategies for the control of fecal–oral pathogens. It has the potential to reduce diarrhea by up to 48%^3^ and reduce acute respiratory infections by up to 27%.^4^

### Current practices and the shortcomings of soap in emergencies.

Despite this, access to hand-washing facilities, water, and soap in humanitarian emergencies is often limited. Those affected by emergencies typically rely on nongovernment organizations (NGOs) to distribute soap and water. However, this approach alone has not been found to increase hand-washing rates.^5,6^ This is partly because when water and soap are scarce, they are prioritized for tasks other than handwashing.^7–9^ Second, it is often difficult for NGOs to meet the demand of beneficiaries—particularly in protracted crises. The Sphere Standards^10^ recommend that in an emergency, one person should have 250 g of bathing soap per month and at least 15 L of water per day. In 2016, 65.6 million people were displaced^11^ from their homes because of conflict, persecution, and crises, and therefore, meeting the needs of all these people was a mammoth logistical task—particularly for a product like soap that is heavy (when ordered in bulk) and requires regular replacement.

In many camp settings, hand-washing and toilet facilities are not designed in ways that make it easy for crisis-affected populations to always wash their hands with soap.^12^ Consider this typical example of a camp setting. Most camps have shared latrines, and alongside these, there are shared hand-washing facilities (although many camps still lack hand-washing facilities altogether). Invariably, the soap that NGOs provide is rarely placed at these shared facilities^5^ because crisis-affected populations worry about it being stolen and used by others. Instead, if individuals wanted to wash their hands with soap after using the toilet, they would be required to walk to the toilet carrying their own bar of soap. On reaching the shared toilet, there is nowhere for these individuals to store the soap hygienically—perhaps it would be placed on the unclean floor. If they were then to wash their hands with soap after using the toilet, then this would mean that they would have to walk back to their shelter with the slimy bar of soap, meaning that their hands would still be covered with soap at the end of the process. Alternatively, people may try to wash their hands within their shelters—bringing contamination into the home environment. In practice, both options are inconvenient and impractical, so hands are normally just rinsed with water or not washed at all.^5^

Soap is thought to have been in existence since 2,800 bc,^13^ and there are records of it being used widely across history and cultures. Although it is an ideal hand-washing product in the vast majority of settings, the particular dynamics of humanitarian crises reduce its practicality and usability.

### The SuperTowel^®^ (ST): a soap alternative.

To overcome some of the limitations of soap, we wanted to develop and test the efficacy of an alternative soap product—the ST. The ST is a product developed by Real Relief (www.realreliefway.com). It is a durable fabric with permanent antimicrobial bonding. The treated fabric must be dipped in water and then rubbed against the hands so that pathogens will be transferred to the fabric, where they will be killed. The antimicrobial technology does not involve toxic chemicals. Instead, it is achieved by long chains of carbon atoms attached to positively charged nitrogen atoms bonded to a silica layer of the fabric. The positively charged layer attracts negatively charged microbes (including bacteria, protozoa, fungi, and encapsulated viruses), causing membrane disruption of the microbes. A fabric with the same antimicrobial bonding has already been tested and used as part of hospital linen and as part of reusable menstrual pads. Tests associated with these products have shown that the antimicrobial treatment was able to reduce 99.9% of *Escherichia coli*, *Staphylococcus*, *Aspergillus brasiliensis*, *Aspergillus niger*, *Pseudomonas aeruginosa*, *Clostridium sporogenes*, and *Klebsiella pneumoniae* and 99.3% of *Candida albicans*.^14^ These tests were based on the fabric directly being spiked with these pathogens. Tests also assessed whether the efficacy of the antimicrobial treatment declined with use, but no such decrease was observed.

The ST offers several potential benefits in emergencies. First, the fabric is durable and the antimicrobial bonding is permanent, so, unlike soap, the ST will not need to be frequently distributed. It will only need replacing when it gets lost or is so visually worn that it is no longer desirable to use. The ST will also be logistically easier to distribute than soap as it is smaller and lighter. It will also be less expensive over time, given its less frequent distribution. The cost per ST unit is currently estimated to be 50 cents. The ST only needs to be dipped in a small amount of water, so it has the potential to dramatically reduce the amount of water used for handwashing. This, in turn, may reduce drainage problems around hand-washing facilities in emergencies, which can otherwise act as vector breeding sites. The ST can also be safely shared between people as the antimicrobial fabric kills 99% of pathogens within 30 seconds.^14^ Importantly, the ST will be beneficial to crisis-affected populations as it can be easily carried by users all the time, making hand cleaning more convenient.

The aim of this study was to assess the efficacy of the ST as a hand-cleaning product and compare it with reference, non-medicated, liquid soap using a controlled laboratory test among healthy volunteers. The laboratory testing and design process described in this study aimed to assess 1) whether the ST could effectively remove microbes from hands and transfer them to the towel and 2) whether hand cleaning with the ST could result in an average log_10_ reduction in pathogens that was equivalent to or better than handwashing with soap.

## METHODS

Two rounds of laboratory testing were conducted on the ST. For the first round, we applied the antimicrobial bonding to a soft satin polyester/cotton fabric, similar to what had been used in hospitals for bed linen. We realized that the satin polyester/cotton fabric was likely to be poor at removing pathogens from hands and was not pleasant to rub on the skin. This led us to explore microfiber fabrics. Some microfiber fabrics have been found to remove 99% of bacteria from surfaces.^15^ In selecting the materials for the second round of testing, we considered the water absorption and feel of the fabric (based on the assumption that the ST would have to feel nice when rubbed on the hands to be used) and explored different textures that could facilitate cleaning under fingernails (because nails are known to harbor pathogens).^16–18^

### Microorganism.

To test the efficacy of the ST, we used an adapted protocol of the European Committee for Standardization (EN 1499)^19^ which is designed to evaluate the ability of hand-wash agents to eliminate transient pathogens from volunteers’ hands without regard to resident microorganisms. This procedure is based on the “post-contamination treatment” of hands and involves the placement of the test organism (*E. coli* [ATCC 11229]) on the hands of test subjects, followed by exposure of the test product.

### Subjects.

The studies were performed in the Department of Microbiology, Kelkar Education Trust’s (KET’s) Scientific Research Centre, Mumbai (India). The first test was conducted in December 2017 and the second set of tests in April 2018. The studies were approved by the London School of Hygiene and Tropical Medicine Ethics Committee and the KET’s Institutional Scientific & Ethics Committee.

Sixteen adult volunteers were selected for each of the laboratory test rounds. Written consent was obtained from all of them. Volunteers were students at nearby universities and were recruited by using poster advertisements that were placed within these nearby institutions. Eligible volunteers had to be male, older than 18 years; must have short fingernails with no artificial nails; must have no cuts or wounds on their hands; must have no history of drug allergies; and must not have taken any antibiotics in the last 2 weeks. All volunteers were physically examined before their involvement in the studies to ensure they were healthy and had healthy skin (people with skin disorders such as eczema, paronychia, psoriasis, scabies, abrasions, lacerations, or skin allergies were excluded). Participants were asked to remove all forms of jewelry from their hands before handwashing. Rings are known to retain bacteria,^20,21^ so removal was necessary so as not to affect the recovery pre- and post-values of the tests.

### New product.

#### First laboratory tests.

The STs used in the tests were produced by Real Relief and measured 25 cm^2^. In the first set of experiments, we tested the original ST (ST0) product. We used a crossover design in which the 16 volunteers were allotted randomly to two groups of the same size. One group applied the new test formulation (ST0) and the other a reference hand-washing product (Johnson & Johnson odor-free liquid soap) using a standardized procedure. In a consecutive run, the two groups reversed roles (crossover design). At the end of the whole series of runs, every subject had used each hand-washing product once.

#### Second laboratory tests.

**Figure 1.**
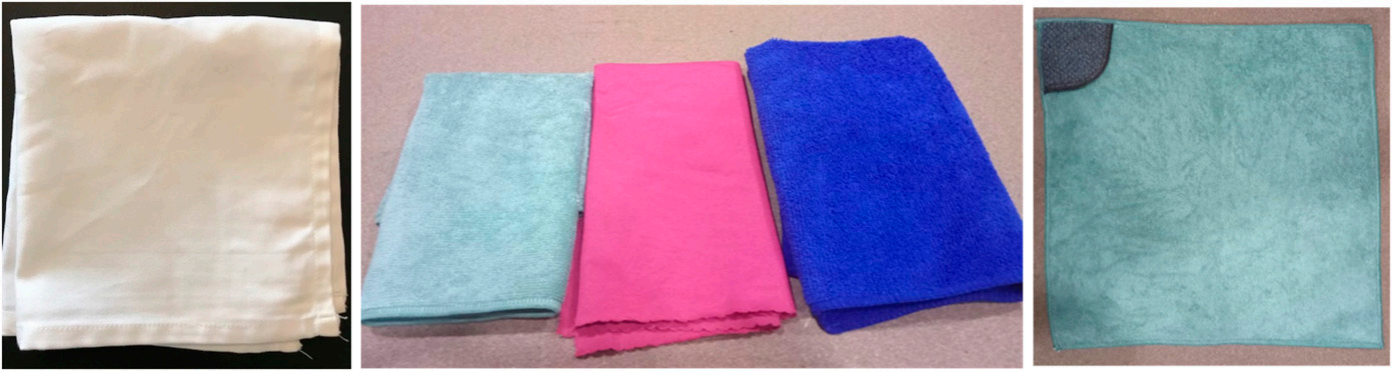
SuperTowel (ST) versions—left picture: ST0; middle picture: ST1, ST2, and ST3; and right picture: ST3 showing the scourer-like material in one corner.

Three new prototypes were created and tested against the same reference soap during the second round of tests. SuperTowel prototype 1 (ST1) was a cloth made out of terry towel fabric. The cloth is a microfiber, composed of 80% polyester and 20% polyamide. SuperTowel prototype 2 (ST2) was a cloth made of thin pique woven material. The cloth’s fiber composition is 80% polyester and 20% polyamide. SuperTowel prototype 3 (ST3) was made from the same material as ST1 but had one corner that was made from a scourer-like material. This was designed to act as a nail scrubbing pad. All the prototypes were treated to have the antimicrobial bonding. Figure 1 shows examples of the ST prototypes.

In the second set of tests, we used a Latin square design where 16 different sequences of the four treatments (ST1, ST2, ST3, and reference soap) were created beforehand. The sequences were allotted to individual volunteers by means of a number draw. This process meant that each volunteer used all of the hand-washing products once and did so in the order prescribed by the randomly selected sequence.

### Contamination procedure.

The hands of each volunteer were washed with a non-medicated soap, dried (thoroughly with paper towels), and immersed up to the mid-metacarpals for 5 seconds with fingers spread apart in a contamination fluid containing nonpathogenic *E. coli* (ATCC 11229) 8.3 × 10^8^ cfu/mL. Excess of fluid was drained off, and the hands were air-dried for 3 minutes.

### Pre-value.

Immediately after drying, the fingertips and thumbs of the left and right hands were rubbed in separate Petri dishes containing 10 mL of tryptone soya broth (TSB) (without neutralizers) for 60 seconds to assess the release of the test organism before treatment of the hands (pre-value). The pre-value was estimated using the standard serial dilution method.^22^

### Hygienic hand-washing procedure.

When testing the reference soap, volunteers were asked to use 5 mL of non-medicated liquid soap and 1 mL of water. They were asked to wash their pre-contaminated hands for 60 seconds by following the “WHO guidelines for handwashing when hands are visibly soiled”^23^ (a diagram of the steps was given to them). Afterward, volunteers were asked to rinse their hands under running tap water for an additional 15 seconds and then air-dry them for 3 minutes (without the use of a machine).

The ST procedure was the same irrespective of the prototype being used. The process involved soaking the ST in water by submersing it completely in a bucket filled with tap water. The amount of water absorbed by the ST was recorded by means of weighing the towel before and after soaking. Participants rubbed their pre-contaminated hands with the wet ST for 60 seconds. They were asked to use steps 2–7 of the “WHO guidelines for how to wash hands when visibly soiled”^23^ as a guide for cleaning their hands with the ST (this excluded the steps related to soap and water). In the case of ST3, the nail-cleaning procedure illustrated in picture 7 of the WHO guideline was performed by rubbing the fingertips on the nail scrubbing pad. Participants were not given guidance on how long they should scrub their nails within the 60 seconds. At the end of the 60 seconds, the volunteers’ hands were allowed to air-dry for 3 minutes (without a machine).

A subsample of participants were video-recorded while completing the tests, and one of the authors (R. K.) was present for all of the tests to maintain standardization and monitor quality.

### Post-values.

When participants’ hands were dry, the fingertips and thumbs of the left and right hands were rubbed in separate Petri dishes containing 10 mL of TSB (without neutralizers) for 60 seconds to assess the release of the test organism after treatment of the hands (post-value). Post-values were determined using the dilution method.^22^ After the procedure, the volunteers were given medicated soap to wash their hands.

### Questionnaire.

At the end of the second study, each volunteer was invited to complete an administered questionnaire, aimed at collecting information about the perceived feasibility of using the ST at different critical times for handwashing and identifying user preferences between the prototypes. The questionnaire was developed by the authors and was designed to provide initial insights into the product’s acceptability. The questions were not related to humanitarian contexts as none of the participants had been personally affected by crises. Instead, they explored the potential use of the ST within the home and in public settings.

### Statistical analysis.

For both the reference soap and the ST products, log_10_ counts from the left and right hands of each subject were averaged separately, for both pre- and post-values. The arithmetic means of all individual log_10_ reduction values were calculated. The statistical analysis was performed with the statistical package STATA version 13.0 (StataCorp., College Station, TX). The distribution of the data was assessed using kurtosis and skewness tests. Given that the data were not normally distributed, nonparametric tests were used. In the first set of experiments, Wilcoxon matched-pair signed-rank test was used to test for the difference between the first ST product and the reference soap. In the second set of experiments, as more than one product was compared, we used the Kruskal–Wallis test, and if evidence of a significant effect was observed, then we used post hoc pair-wise tests (Wilcoxon signed-rank tests) to assess the differences between specific pairs. The new product was considered to have the same efficacy as the reference product (soap) if the mean log_10_ reduction factor was not significantly smaller for the former than for the latter. Because of the confirmative nature of the test on this application, the level of significance is set at *P* = 0.05 and the test used is two-sided. The discrimination efficiency of the test procedure described has been set to detect a difference between the two mean log_10_ reduction factors of approximately 0.6 log_10_ at a power of 95%. This results in a sample size of *N* = 16 for each set of experiments.

## RESULTS

A total of 32 people participated in the tests: 16 in the first set of experiments and 16 in the second one. All were male, lived in an urban area, and ranged in age from 18 to 43 years (average age 21 years).

**Figure 2.**
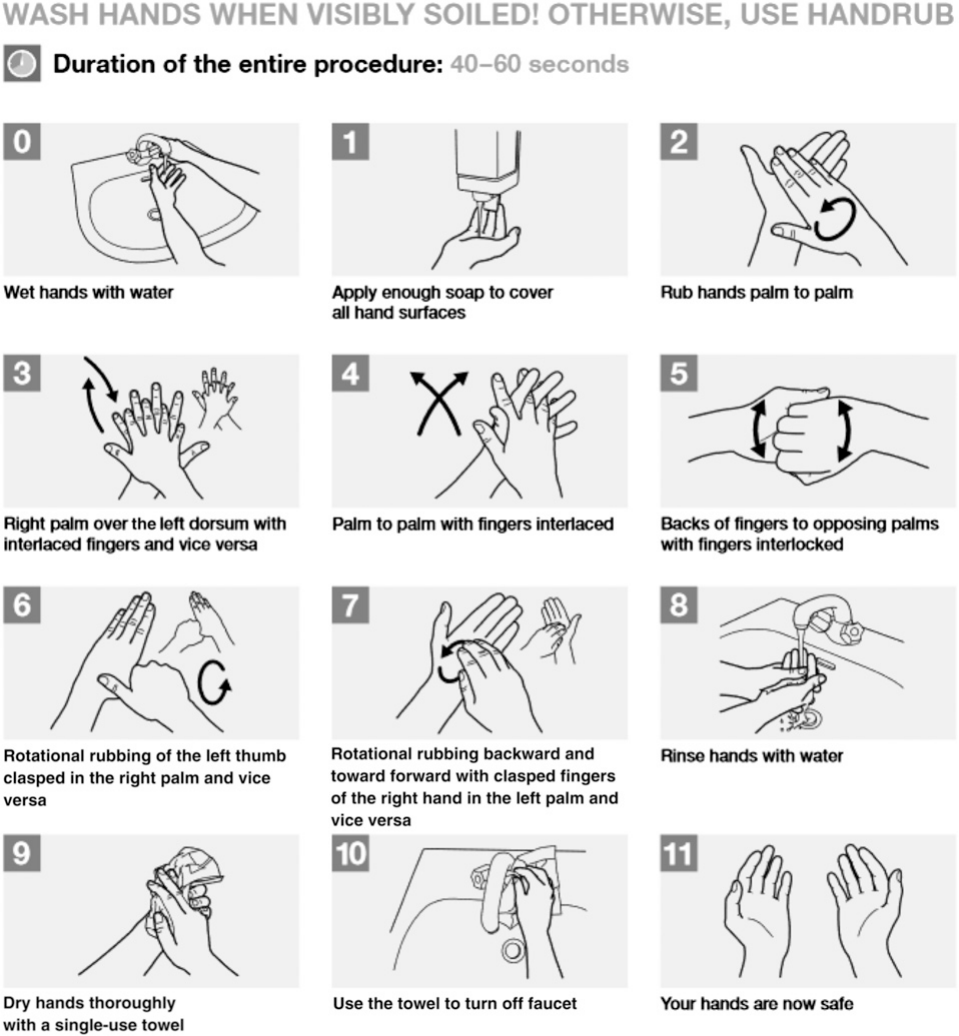
Directions for handwashing provided to participants in both rounds of laboratory testing.

**Figure 3.**
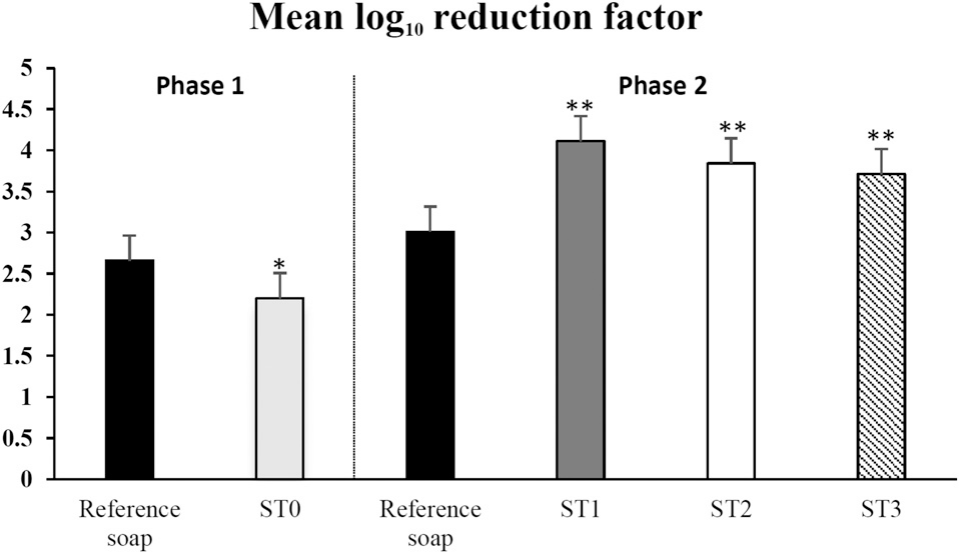
Mean log_10_ reduction factor of *Escherichia coli* after volunteers washed their hands with either the SuperTowel prototypes or the reference product (non-medicated soap). (Left side, phase 1 experiment, *P = 0.018. Right side, phase 2 experiment, **P < 0.005).

Table 1 and Figure 2 describe the results from both sets of experiments. The overall mean of the log_10_ pre-values was 7.6, and the maximum detectable log_10_ reduction observed in these experiments was 4.95. In the first set of experiments, we observed that use of the ST0 prototype resulted in a mean log_10_ reduction of 2.20 ± 0.57. For ST0, the reduction observed was significantly less effective than that with the reference soap (2.66 ± 0.42, *P* = 0.02). In the second set of tests, we observed differences in log_10_ reduction with the different ST products (Kruskal–Wallis *P*-value = 0.0001). A mean log_10_ reduction of 4.11 ± 0.47 for ST1, 3.84 ± 0.61 for ST2, and 3.71 ± 0.67 for ST3 was detected, which was significantly higher than that observed with the reference soap of 3.01 ± 0.63 (*P* < 0.001, *P* = 0.002, and *P* = 0.005, respectively). SuperTowel prototype 1 was more efficient in reducing bacteria than ST3 (difference in log_10_ reduction = 0.4, *P* = 0.03). The bacterial reductions from all tests are shown in Figure 3.

**Table 1:** Mean log_10_ reduction factor of *Escherichia coli* after volunteers washed their hands with either the SuperTowel (ST) prototypes or the reference product (non-medicated soap)

Product	Mean log_10_ reduction factor for ST (SD)	Mean log_10_ reduction factor for reference soap (SD)	Difference	*P*-value*
Phase 1 test				
ST original	2.20 (0.57)	2.66 (0.42)	0.46	0.018
Phase 2 tests				
ST prototype 1	4.11 (0.47)	3.01 (0.63)	1.1	0.001
ST prototype 2	3.84 (0.61)	3.01 (0.63)	0.83	0.003
ST prototype 3	3.71 (0.67)	3.01 (0.63)	0.7	0.005

* *P*-values were derived using Wilcoxon’s matched-pair signed-rank tests, SD.

The process of hand rinsing for 15 seconds after handwashing with the reference soap consumed 1.2 L of water. The ST prototypes absorbed on average 36 ± 9 mL (ST0), 318 ± 14 mL (ST1), 80 ± 4 mL (ST2), and 171 ± 15 mL (ST3) of water.

In the questionnaire, the 32 participants were asked to compare the reference soap with the STs. These results are summarized in Table 2. Participants felt that the STs would allow them to wash their hands more frequently than soap. People felt that the STs would be particularly useful if they needed to clean their hands when they were away from home. The 16 participants who participated in the second study were also asked to compare prototypes ST1, ST2, and ST3. Participants indicated that ST3, with the “nail pad,” was their preferred prototype, but five people preferred ST1 (none of the participants chose ST2 as their preferred ST, and one person did not respond to this question).

**Table 2:** Questionnaire responses comparing the SuperTowel (ST) products to the reference soap

Question	Any of the STs used in the trial (*N* = 32)	Soap used in the trial (*N* = 32)
If you owned both products, which would enable you to wash your hands more frequently?	24	8
Which of the products would you prefer to use after defecation/after going to the toilet while at home?	13	19
Which of the products would you prefer to use after defecation/after going to the toilet in a public place?	27	5
Which of the products would you prefer to use before eating at home?	17	15
Which of the products would you prefer to use before eating outside of the home?	28	4

## DISCUSSION

The first ST prototype (ST0) showed less efficacy in reducing bacteria from artificially contaminated hands than soap. Three new microfiber prototypes of ST were designed, and all showed better efficacy in removing bacteria than soap. SuperTowel Prototype 1 was the most efficacious.

Through our design workshop, we identified that the smoothness of the ST0 fabric (made from a satin cotton/polyester blend) probably prevented pathogens from being transferred from the hands to the towel. This led us to explore and include microfiber fabrics in the second round of testing. The second set of laboratory tests found that all three of the microfiber ST prototypes were more efficacious than handwashing with soap. Microfibers are produced by combining many very thin polyester and polyamide fibers into a single fiber during production. However, unlike conventional cloths, they have thousands of randomly arranged sharp-edged microfiber strands, which improve cleaning efficacy.^24^

Different studies have found that mechanical friction applied during handwashing plays a role in removing microorganisms adhering to the hands.^25^ This could explain why three of the ST prototypes were able to remove more bacteria from volunteers’ hands than soap. The added advantage of the ST in comparison with other hand-wiping materials is that the antimicrobial bonding technology ensures pathogens landing on the fabric are destroyed because of membrane disruption.

The most effective prototype was ST1, which was made from a relatively thick and soft terry towel fabric. SuperTowel prototype 3 was made from the same fabric but included a corner made from scourer-like fabric, designed for cleaning nails. Although ST3 was found to be more efficacious than handwashing with soap, it was the least effective of the three microfiber prototypes. By re-watching the video recordings, we identified that when using ST3, participants prioritized scrubbing their nails on the pad and did not allocate as much of the 60 seconds to wiping the cloth over the rest of their hands. This likely explains the lower rates of bacteria removal. Although participants preferred using the ST3 prototype, these observed patterns of use and its reduced efficacy suggest that ST1 is a more reliable product.

All four versions of the ST used much less water than handwashing with soap, indicating its potential usefulness in water-scarce settings. The questionnaire responses indicated that the ST would be preferable to soap in public environments or when outside the home. This supports the hypothesized use of this product during or in the wake of humanitarian crises because facilities are commonly shared or not always available. It also suggests that the product could be appealing in noncrisis settings, whenever handwashing with soap is not desirable or convenient.

There were several limitations of our study. To be comparable with other studies, the tests followed an adapted version of the European Committee Standard for evaluating hand antiseptic agents. However, these standards are principally designed for assessing the efficacy of new hand-wash or hand-rub agents to be used in health-care settings. At present, there is a lack of clarity about whether this study protocol is also appropriate for domestic-focused efficacy studies like this one. The same protocol has previously been used to test the efficacy of *Moringa oleifera* plant powder as a hand-cleaning product for potential use in domestic settings.^26^ We also do not know what size of bacterial log_10_ reduction would have a meaningful public health impact in domestic settings.^27,28^ A further limitation of this European Committee Standard is that it requires 60 seconds of hand cleaning with the reference soap and each of the test products. Commonly, in real-world settings, people wash their hands for just 5–10 seconds.^29^

The studies described here provide a useful proof of concept, under laboratory conditions, but further testing could be carried out to explore the comparable efficacy of the ST when used for a more realistic hand-washing duration or on hands that were naturally contaminated. It is likely that future users of the ST would have to carry it around on their person. Therefore, there would be value in assessing whether the product can achieve comparable efficacy if used when moist rather than soaking wet. Third, it would be interesting to assess whether the ST can achieve equivalent efficacy if a person’s hands are visibly dirty (e.g., covered in mud) or oily. Given the process by which the antimicrobial treatment kills pathogens, it is biologically plausible that the ST could be used with water of any quality (e.g., gray water). However, it would be useful to verify this through laboratory-based efficacy tests with a range of water qualities and field-based acceptability studies to determine whether people would feel comfortable with gray water being used for hand cleaning in this way.

For practical reasons, we excluded women from our study. Indian regulations require that if any new product is to be tested on women, then they must undergo pregnancy screening. We did not want to subject female participants to this and, therefore, decided to have male participants only. The lack of female participants is unlikely to have had an impact on the primary results, given that hand cleaning was performed in a prescribed and standardized way during these studies. However, scientific research should always aim to include women equally.^30,31^ For studies to do with hygiene, this is important because of gendered differences in real-world hand-washing practices (women tend to wash their hands more frequently and more thoroughly than men)^32–39^ and because in the Indian context, women are primarily responsible for preparing food and may also have different attitudes toward handwashing after defecation. In this study, it would have been particularly useful to get the opinions of women as part of the questionnaires.

The antimicrobial treatment used on the ST can effectively kill all bacteria, fungi, and protozoa; however, it only kills viruses that are encapsulated. It is likely that the microfiber material used in the ST will remove diarrhea-causing nonencapsulated viruses (such as norovirus and rotavirus) and bacterial spores (such as those produced by different strains of *Clostridium*) from hands and transfer them to the ST without killing them (although this would need to be substantiated through further laboratory testing). This inability to kill nonencapsulated viruses is not unique to the ST. For example, norovirus, rotavirus, and *Clostridium difficile* have been found to be resistant to typical infection control measures (such as alcohol-based sanitizers) and persist in the environment and on hands and surfaces for a long time.^40–42^

The future uptake of the ST will rely on its acceptability among crisis-affected populations. To encourage a population who are used to handwashing with soap to use a ST will require a substantial behavioral shift. It is envisaged that the form and branding of the product may have to be improved to facilitate the product’s intuitive use. This is something that will require field-based testing and input from crisis-affected communities. A field study with STs in a refugee camp in the Tigray region of Ethiopia is already scheduled as a next step in this project and will be the subject of a further scientific study.

## CONCLUSION

These studies have demonstrated that under controlled conditions, the microfiber prototypes of the ST were more efficacious at removing nonpathogenic *E. coli* from pre-contaminated hands than handwashing with soap. SuperTowel prototype 1 was found to be the most efficacious and was relatively well liked by participants. These findings, when taken together with the product’s prior testing results related to the antimicrobial bonding treatment of other fabrics, provide a promising indication of the ST’s potential to be used as an alternative or complementary hand-cleaning product. The ST was designed to be a product suited to hand cleaning in difficult circumstances (such as humanitarian crises) where handwashing with soap is not a feasible, convenient, or desirable option. Our findings indicate that the ST is appropriate for use in contexts where soap and water availability is scarce or irregular, and where sanitation and hygiene facilities are not available or are shared and, therefore, considered less desirable to use. Given that the ST is more lightweight than soap and longer lasting, we envisage that the ST would also be beneficial to humanitarian actors working in areas where logistical and security issues make regular hygiene product distribution challenging. We recommend that further testing should be carried out to assess the efficacy of the ST under conditions that more closely mirror real-world hand-washing practices. Further development of the ST should be carried out in consultation with crisis-affected populations to ensure the product meets their needs.
